# Immunosuppressive Compounds Affect the Fungal Growth and Viability of Defined *Aspergillus* Species

**DOI:** 10.3390/pathogens8040273

**Published:** 2019-11-29

**Authors:** Stanislaw Schmidt, Michael Hogardt, Asuman Demir, Frauke Röger, Thomas Lehrnbecher

**Affiliations:** 1 Division of Pediatric Hematology and Oncology, Hospital for Children and Adolescents, University Hospital, Goethe University Frankfurt am Main, Theodor-Stern-Kai 7, 60590 Frankfurt, Germany; stanislaw.schmidt@kgu.de (S.S.); asuman.demir@kgu.de (A.D.); frauke.roeger@kgu.de (F.R.); 2 Institute of Medical Microbiology and Infection Control, University Hospital, Goethe University Frankfurt am Main, Theodor-Stern-Kai 7, 60590 Frankfurt, Germany; michael.hogardt@kgu.de

**Keywords:** *Aspergillus* species, immunosuppressive agent, mycophenolic acid, methylprednisolone, cyclosporin A, growth inhibition, viability

## Abstract

Immunosuppressive drugs are administered to a number of patients; e.g., to allogeneic hematopoietic stem cell transplant recipients. Immunosuppressive drugs impair the immune system and thus increase the risk of invasive fungal disease, but may exhibit antifungal activity at the same time. We investigated the impact of various concentrations of three commonly used immunosuppressive compounds—cyclosporin A (CsA), methylprednisolone (mPRED), and mycophenolic acid (MPA)—on the growth and viability of five clinically important *Aspergillus* species. Methods included disc diffusion, optical density of mycelium, and viability assays such as XTT. MPA and CsA had a species-specific and dose-dependent inhibitory effect on the growth of all *Aspergillus* spp. tested, although growth inhibition by MPA was highest in *A. niger,* *A. flavus* and *A. brasiliensis*. Both agents exhibited species-specific hyphal damage, which was higher when the immunosuppressants were added to growing conidia than to mycelium. In contrast, mPRED increased the growth of *A. niger*, but had no major impact on the growth and viability of any of the other *Aspergillus* species tested. Our findings may help to better understand the interaction of drugs with *Aspergillus* species and ultimately may have an impact on individualizing immunosuppressive therapy.

## 1. Introduction

Immunosuppressive drugs have to be administered in a number of clinical conditions; e.g., in patients with autoimmune diseases such as rheumatoid arthritis, psoriasis or autoimmune hemolytic anemia, in patients after renal, liver or heart transplantation to prevent graft rejection or in allogeneic hematopoietic stem cell transplant (HSCT) recipients in order to prevent or to treat graft-versus-host disease (GvHD). Depending on the indication, the compounds are administered at different dosages and schedules. Immunosuppressive drugs exhibit specific negative effects on the different arms of the immune system [1]. For example, glucocorticosteroids such as prednisolone or methylprednisolone (mPRED) not only suppress the phagocytic function of monocytes and neutrophils, but also impair antigen presentation, T cell function, and the expression of pro-inflammatory cytokines [2,3]. Mycophenolate mofetil (MMF), which is metabolized after administration to its active compound mycophenolic acid (MPA), impairs the recruitment of monocytes and lymphocytes into sites of inflammation and induces the apoptosis of activated T lymphocytes [4,5]. The calcineurin inhibitor cyclosporin A (CsA), another commonly used immunosuppressive agent, is a fungus-derived molecule and inhibits relatively selective T cell activation, whereas the compound has a minimal effect on phagocytic cells [6]. Although it is well described that the impairment of the host immune system by immunosuppressive drugs increases the risk of invasive fungal disease [7,8], there are data indicating that some immunosuppressive compounds exhibit activity against *Aspergillus fumigatus*, *Candida albicans* or *Cryptococcus neoformans* [9,10]. *Aspergillus* spp. is the most frequent cause of invasive fungal disease in patients with hematological malignancies or undergoing HSCT. As *A. fumigatus*, *A. brasiliensis*, *A. terreus*, *A. flavus*, and *A. niger* comprise up to 70% of *Aspergillus* species isolated in HSCT patients suffering from invasive fungal disease [11], and *A. flavus* and *A. niger* often have reduced susceptibility to amphotericin B, which may affect outcomes [12], we thought to evaluate the effect of three commonly used immunosuppressive compounds—mPRED, MPA, and CsA—on these distinct species of *Aspergillus.*

## 2. Results

### 2.1. Immunosuppressive Agents Inhibit the Fungal Growth of Aspergillus Species—Disc Diffusion Assay for Screening 

**Figure 1:**
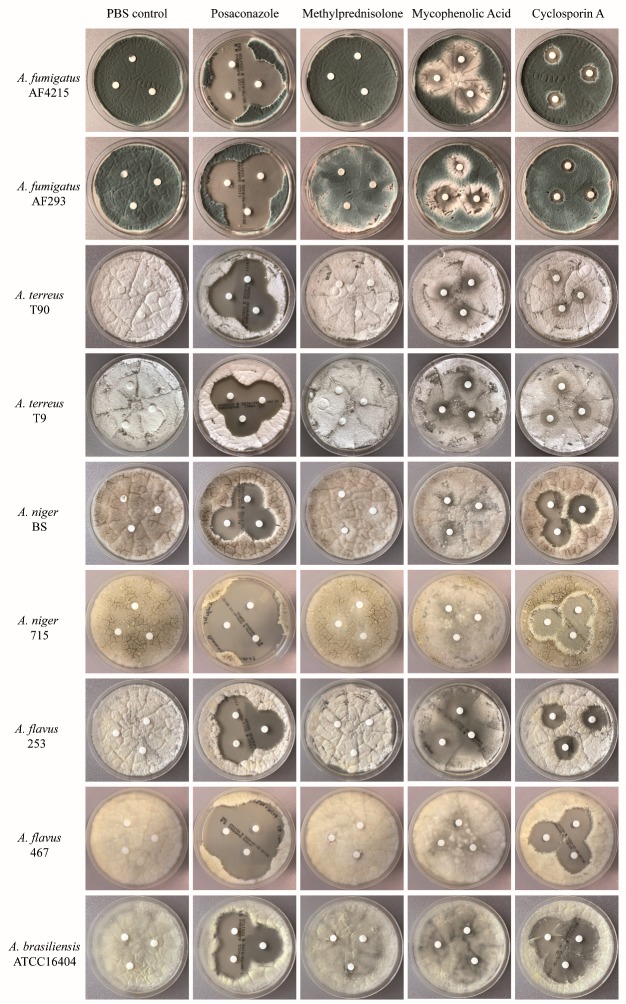
Effect of immunosuppressive compounds on the growth of *Aspergillus* spp. Three 6 mm diameter paper discs were impregnated with 35 µL of a 16 mg/mL methylprednisolone (mPRED) solution, a 50 mg/mL mycophenolic acid (MPA) solution, a 50 mg/mL cyclosporin A (CsA) solution, a 0.5 µg/mL posaconazole solution, or PBS, respectively, and placed onto inoculated agar plates. Shown are representative results of one test; the assay was performed three times for each fungus and for each immunosuppressive drug at each concentration investigated.

The disc diffusion assay was used as a screening method to determine whether any of the immunosuppressive compounds had an impact on the growth of the *Aspergillus* species included in the study. The assay revealed that MPA and CsA exhibited an inhibitory effect on the growth of all *Aspergillus* species tested, although the extent of inhibition differed between the species (Figure 1). Growth inhibition by MPA was considerably more pronounced for *A. flavus* strain 253 and *A. brasiliensis* compared to both strains of *A. fumigatus*, *A. terreus* and *A. niger*, respectively. Similarly, CsA inhibited the growth of *A. niger*, *A. flavus* and *A. brasiliensis* to a higher extent compared to *A. fumigatus* and *A. terreus*, respectively. In contrast, mPRED had a marginal inhibitory effect on the growth of *A. niger*, but no impact on the growth of any of the other *Aspergillus* species tested was observed. Growth inhibition by all the immunosuppressive agents was lower compared to that of posaconazole. Of note is that the immunosuppressive drugs used at very high concentrations inhibited the growth of the fungi on the agar plates, whereas no major effect in the disc assay was seen when the immunosuppressive compounds were tested in lower concentrations (data not shown).

### 2.2. Immunosuppressive Agents Inhibit the Fungal Growth of Aspergillus Species—Quantitative Assessment of Fungal Cell Density 

**Figure 2:**
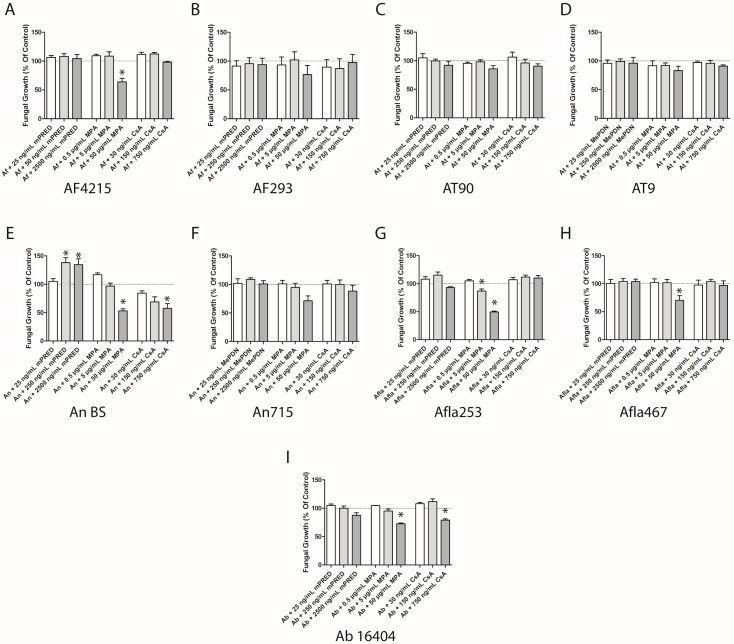
Immunosuppressive compounds at high concentrations reduce the fungal growth of *Aspergillus* spp. Various concentrations of immunosuppressive compounds were added to resting conidia for 17 hours, and the cell density of *A. fumigatus* (**A**,**B**), *A. terreus* (**C**,**D**), *A. flavus* (**E**,**F**), *A. niger* (**G**,**H**) and *A. brasiliensis* (**I**) mycelium was assessed. Shown are the mean and SEM of at least three independent experiments. Ab—*Aspergillus brasiliensis*; Af—*A. fumigatus*; Afla—*A. flavus*; An—*A. niger* complex (*A. niger*); At—*A. terreus*; mPRED—methylprednisolone; MPA—mycophenolic acid; CsA—cyclosporin A. *: *p* < 0.05.

In order to further evaluate and quantify the observations made in the disc diffusion assay, we determined the impact of the immunosuppressive agents on the growth and germination of resting conidia of the different *Aspergillus* spp. in yeast nitrogen base (YNB) medium by the assessment of fungal cell density. In this assay, three different concentrations of the immunosuppressive compound were investigated, with a concentration reflecting common target serum levels, a significantly lower and a significantly higher concentration. Fungal cell density was assessed after 17 hours of incubation of resting conidia with the respective immunosuppressive drug. Compared to untreated controls, MPA at a concentration of 50 µg/mL significantly suppressed the formation of mycelium of *A. fumigatus* AF4215, *A. brasiliensis*, both *A. flavus* strains tested and *A. niger* strain BS for up to 50% (*p* < 0.05 each; Figure 2A,E,G–I), respectively, whereas *A. fumigatus* AF293, both *A. terreus* strains tested (Figure 2C,D) and *A. niger* 715 (Figure 2F) were not significantly affected. With the exception of *A. flavus* strain 253 (Figure 2G), lower concentrations of MPA did not result in a significant reduction of the cell density of any of the species tested. Cyclosporin A inhibited the formation of mycelium of *A. niger* strain BS in a dose-dependent manner (mean ± SEM: 84.4% ± 3.8%, (not significant); 68.7% ± 9.1%, (not significant); 57.4% ± 8.4%, *p* = 0.037; Figure 2E), whereas no significant effect was seen on *A. niger* strain 715 (Figure 2F). The highest concentration of CsA significantly reduced the cell density of *A. brasiliensis* (mean ± SEM: 79.0% ± 2.1%, *p* = 0.01; Figure 2I), whereas none of the other *Aspergillus* species tested was affected by the compound at any concentration. In contrast to MPA and CsA, mPRED increased the growth of *A. niger* strain BS (mean ± SEM: 104.9% ± 5.2%; ns, 137.9% ± 8.8%; *p* = 0.0191, 134.5% ± 10.2%; *p* = 0.0442; Figure 2E), but had no major impact on the growth of any of the other *Aspergillus* species analyzed.

### 2.3. Immunosuppressive Agents Damage Aspergillus Species 

**Figure 3:**
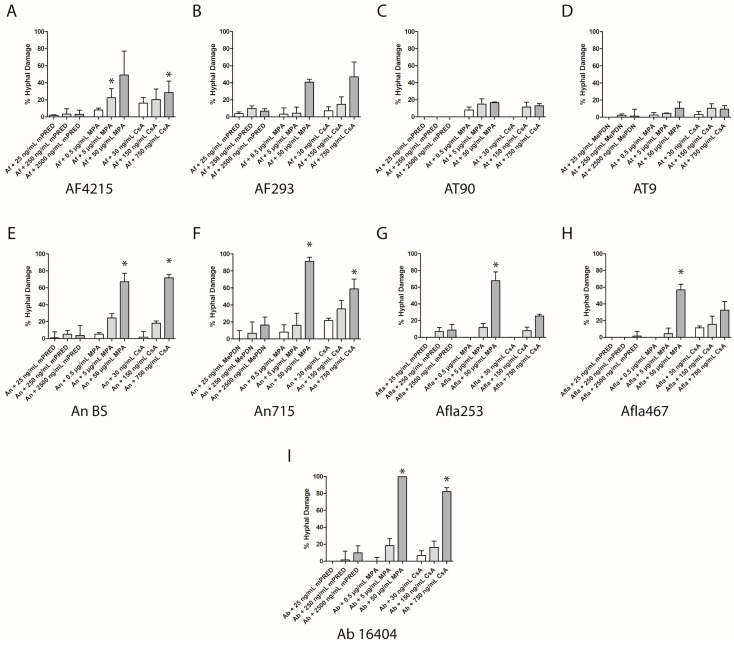
Immunosuppressive compounds reduce the viability of *Aspergillus* hyphae developing from resting conidia. Measured hyphal damage of *A. fumigatus* (**A**,**B**), *A. terreus* (**C**,**D**), *A. flavus* (**E**,**F**), *A. niger* (**G**,**H**) and *A. brasiliensis* (**I**) when various concentrations of immunosuppressive compounds were added to resting conidia for 17 hours. Shown are the mean and SEM of at least three independent experiments. Ab—*Aspergillus brasiliensis*; Af—*A. fumigatus*; Afla—*A. flavus*; An—*A. niger* complex (*A. niger*); At—*A. terreus*; mPRED—methylprednisolone; MPA—mycophenolic acid; CsA—cyclosporin A. *: *p* < 0.05.

In addition to a reduction in the formation of mycelium observed in the disc diffusion assay and the assessment of fungal cell density, both MPA and CsA impaired the viability of developing *Aspergillus* hyphae in a dose-dependent manner (Figure 3). In contrast, for mPRED, only negligible effects were observed. The lowest cell viability, equivalent to the highest damage of developing hyphae, was seen when MPA at the highest concentration was co-incubated with conidia of both strains of *A. niger,* both strains of *A. flavus*, and *A. brasiliensis*, respectively. Similar results were observed for the damage by CsA at a concentration of 750 ng/mL on developing hyphae of both strains of *A. fumigatus*, both strains of *A. niger* and *A. brasiliensis*, respectively (Figure 3A,B,E,F,I). Clinically relevant concentrations of MPA and CsA resulted in a fungal damage of approximately 20% in *A. fumigatus* AF4215, both strains of *A. niger*, and *A. brasiliensis*, respectively (Figure 3A,E,F,I). The lowest hyphal damage of MPA and CsA was seen for both strains of *A. terreus* (Figure 3C,D). 

**Figure 4:**
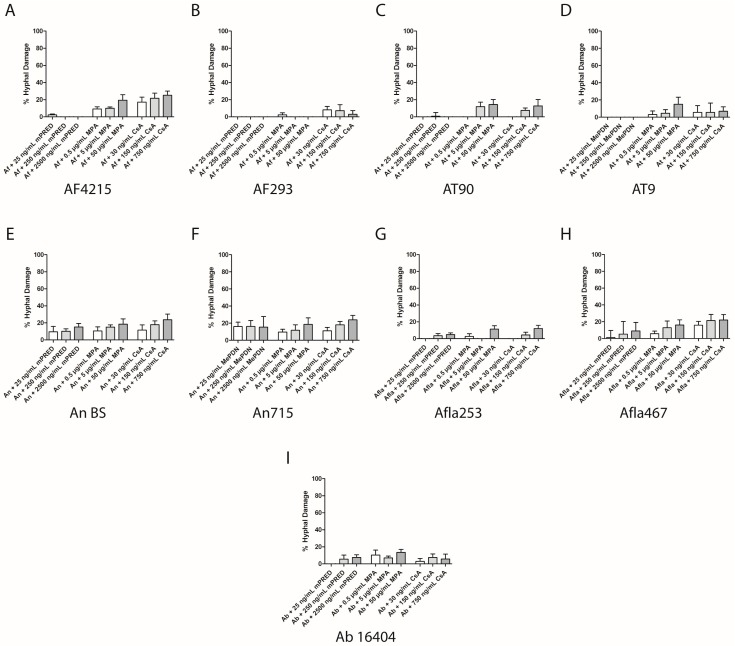
Immunosuppressive compounds decrease the viability of fully developed *Aspergillus* hyphae/mycelium. Measured hyphal damage of *A. fumigatus* (**A**,**B**), *A. terreus* (**C**,**D**), *A. flavus* (**E**,**F**), *A. niger* (**G**,**H**) and *A. brasiliensis* (**I**) when various concentrations of immunosuppressive compounds were added to *Aspergillus* hyphae for 6 hours. Shown are the mean and SEM of at least three independent experiments. Ab—*Aspergillus brasiliensis*; Af—*A. fumigatus*; Afla—*A. flavus*; An—*A. niger* complex (*A. niger*); At—*A. terreus*; mPRED—methylprednisolone; MPA—mycophenolic acid; CsA—cyclosporin A.

When comparing the effect of MPA and CsA co-incubated with *Aspergillus* conidia, less hyphal damage was observed when the immunosuppressive agents were added to *Aspergillus* mycelium for 6 hours. For example, the hyphal damage of *A. niger* BS by MPA and CsA was significantly lower compared to the hyphal damage of the respective agent towards germinating *Aspergillus niger* conidia (mean ± SEM: 18.5% ± 6.2% versus 67.2% ± 10.0% for MPA at a dosage 50 µg/mL (*p* = 0.0144), and 23.6% ± 6.8% versus 71.4% ± 4.0% for CsA at a dosage of 750 ng/mL (*p* = 0.0038); Figure 4E). Similar results were seen for *A. brasiliensis* (mean ± SEM: 13.4% ± 3.6% versus 99.8% ± 0.03% for MPA at a dosage 50 µg/mL (*p* = 0.0005), and 5.7% ± 5.8% versus 82.3% ± 4.6% for CsA at a dosage of 750 ng/mL (*p* < 0.0001); Figure 4I). With the exception of *A. niger*, mPRED showed minimal to no damage towards all other *Aspergillus* species tested (Figure 4). In order to directly compare the fungal damage of resting conidia incubated with immunosuppressive compounds for 17 hours, we extended the incubation time of the mycelium with immunosuppressive compounds from 6 to 17 h. The extended incubation time did not result in an increased hyphal damage compared to 6 hours (Supplementary Figure S1).

## 3. Discussion

Our data demonstrate for the first time that various immunosuppressive compounds exhibit specific activities on the growth and viability of different species of *Aspergillus*, the most common cause of invasive fungal infection. Although an early microscopic analysis revealed that CsA delays the filamentation of *A. fumigatus* [13], which has also been described for other fungi such as *Neurospora crassa* and *Cryptococcus neoformans* [14,15], data on the effect of CsA on other important *Aspergillus* species such as *A. terreus* or *A. niger* were lacking. Our data assessing fungal growth and viability show that CsA exhibits antifungal activity across all *Aspergillus* species tested, although the effect was most pronounced in *A. niger* and *A. brasiliensis*. In addition, higher concentrations of CsA mostly had a stronger effect than lower concentrations, although clinically relevant concentrations of CsA resulted in the damage of approximately 20% of *A. fumigatus*, *A. niger*, and *A. brasiliensis*, respectively. It has been shown previously that CsA inhibits the growth and hyphal elongation of *A. fumigatus* in concentrations of 625 ng/mL and higher, which are higher than the recommended dosages for clinical use [16,17]. The antifungal activity of calcineurin inhibitors such as CsA is not surprising, as it is known that *Cryptococcus neoformans* requires calcineurin for hyphal elongation during mating [14], and the calcineurin-mediated signal transduction pathway impacts the fungal cell membrane via the regulation of the biosynthesis of ergosterol, chitin and β-glucan [18]. Similar to CsA, we observed an effect of MPA on growth and damage on all *Aspergillus* species tested, with a species-dependent magnitude of the effect. Our data clearly show that, with the exception of *A. terreus,* hyphal damage by MPA was more pronounced when the immunosuppressants were added to growing conidia than to mycelium, suggesting that fully developed hyphae might be less susceptible to immunosuppressants than growing conidia. Although the antifungal mechanism of MPA has not been revealed to date, animal data demonstrate an anti-*Pneumocystis jirovecii* effect of MPA, which is supported by clinical association studies [19,20,21]. In contrast to CsA and MPA, mPRED increased the growth of *A. niger*, but did not have a major impact on the other *Aspergillus* species tested. An early study described a species-specific effect of hydrocortisone, which increased the growth rate of *A. fumigatus* and *A. flavus*, but did not affect *A. niger* and *A. oryzae* [22]. Whether corticosterone-binding proteins—which have been described for various Candida species [23]—are involved in the growth-promoting effect of hydrocortisone in *A. fumigatus* and *A. flavus* is unclear to date. The fact that no impact of corticosteroids on the growth or cell density of *R. oryzae* has been found supports the existence of fungus-specific differences in the effect of corticosteroids on fungal biology [24].

We observed only minor differences between the strains of specific species, although results regarding growth inhibition and viability within one species were comparable. We acknowledge the fact that we tested only a limited number of *Aspergillus* species and strains, and it might be possible that other strains may show different results. It is also important to note that we used concentrations above the pharmacologically relevant dosages for the disc assay, in which the conidia are exposed to different concentrations of the drug, whereas in the experiments assessing OD, all conidia are exposed to an identical concentration of the respective compound. However, no *in vitro* experiment can exactly reflect physiological conditions, and under physiological conditions, even significantly lower concentrations of the compound may have the same biological effect as very high concentrations in an *in vitro* assay. 

Although our data demonstrate that immunosuppressive agents may inhibit the growth and decrease the viability of defined species of *Aspergillus*, it is important to note that the data clearly show that the antifungal activity of the agents do not outweigh their immunosuppressive effects. In this regard, daily injections of CsA could not rescue cyclophosphamide-immunosuppressed mice suffering from lethal pulmonary aspergillosis [16]. Similarly, corroborating a report on MMF [25], the administration of CsA was associated with a four-fold risk of invasive fungal infection within the first 6 months after kidney transplantation compared to corticosteroid and azathioprine therapy [26]. On the other hand, potent synergisms in the activity against a variety of fungi has been described for CsA when combined with azoles, which affected even azole resistant isolates [9,10,27,28]. In conclusion, our data show that 1) MPA and CsA exhibit a species-specific inhibitory effect on the growth *Aspergillus*, 2) higher fungal damage is observed when MPA and CsA are added to growing conidia than to mycelium, and 3) mPRED increases the growth of *A. niger*, but has no major impact on the growth and viability of the other *Aspergillus* species tested. Further studies including microscopic and phenotypic analyses are warranted to reveal the mechanisms of how immunosuppressive agents affect different species of *Aspergillus*, which might not only increase our understanding of the complex interaction of drugs with various *Aspergillus* species but also of the unique characteristics of each immunosuppressive agent (immunosuppression versus antifungal activity), which ultimately may have an impact to individualize immunosuppressive therapy.

## 4. Materials and Methods

### 4.1. Preparation of Fungi 

Two different strains each of *A. fumigatus* (strains AF4215, ATCC MYA 1163 and AF293), *A. terreus* (clinical isolates T9 and T90, provided by Cornelia Lass-Flörl, Medical University of Innsbruck, Innsbruck, Austria), *A. niger* complex (*A. niger*, clinical isolates BS and 715) and *A. flavus* (clinical isolates 253 and 467), and one strain of *A. brasiliensis* (strain ATCC 16404) were grown on Sabouraud glucose agar plates (BD Bioscience, San Jose, CA, USA) at 37 °C for 3 days. Conidia were harvested by gently scraping the surface of the plates, washed in Hanks’ balanced saline solution (Gibco, Paisley, UK) and filtered through sterile gauze. The number of the conidia was estimated in a Neubauer chamber (LO–Laboroptik, Friedrichsdorf, Germany). Resting conidia were used immediately for the experiments or stored at 4 °C for a maximum of one week. For the preparation of *Aspergillus* hyphae, resting conidia were plated in flat-bottom cell culture plates (Nunc, Langenselbold, Germany) and incubated in Yeast Nitrogen Base (YNB; Sigma-Aldrich, Taufkirchen, Germany) medium at 37 °C for 17 h to allow formation of hyphae.

### 4.2. Immunosuppressive Agents 

The immunosuppressive compounds cyclosporin A (CsA; Novartis Pharma, Nürnberg, Germany), methylprednisolone (mPRED; Sanofi Aventis, Frankfurt, Germany), mycophenolic acid (MPA; Sigma-Aldrich, Steinheim, Germany), the active component of mycophenolate mofetil (MMF), were commercially obtained. All agents were stored and dissolved according to the manufacturers´ instructions. Three concentrations were tested for each compound; in addition to the concentration reflecting common target serum levels (e.g., 150 µg/µL for CsA), each compound was also assessed in a significantly lower and higher concentration, respectively. Specifically, CsA was used at concentrations at 0.03, 0.15 and 0.75 µg/mL, MPA at 0.5, 5 and 50 µg/mL, and mPRED at 0.025, 0.25 and 2.5 µg/mL, respectively [3,17,29,30]. Higher concentrations of CsA (50 mg/mL), MPA (50 mg/mL) and mPRED (16 mg/mL), respectively, were used for disc diffusion assay.

### 4.3. Assessment of the Antifungal Activity of Immunosuppressive Agents

The antifungal activity of immunosuppressive agents was tested by assessing growth inhibition by means of disc diffusion assay and by determination of the cell density. The viability of the fungus was assessed by using the XTT assay.

#### 4.3.1. Disc Diffusion Assay 

For the assessment of growth inhibition by the immunosuppressive compounds, disc diffusion assays were performed on agar plates as described elsewhere with some modifications [9]. In brief, 200 µL of a suspension containing 5 × 10^6^ conidia/mL of the fungal strain were spread uniformly onto Sabouraud glucose agar plates (BD Bioscience). Paper discs of 6 mm in diameter (Becton Dickinson) were impregnated with 35 µL of a 16 mg/mL mPRED solution, a 50 mg/mL MPA solution, a 50 mg/mL CsA solution or a 0.5 µg/mL posaconazole (MSD/Merck, Whitehouse Station, NJ, USA) solution, respectively, and placed onto the inoculated agar plates. Plates were then incubated at 37 °C and evaluated for the degree of growth inhibition after 24 h.

#### 4.3.2. XTT Assay 

The effect of the immunosuppressive compounds on the viability of *Aspergillus* hyphae was analyzed by means of the colorimetric XTT assay using (2,3-bis[2-methoxy-4-nitro-5sulphenyl]2H-tetrazolium-5-carboxyanilide) sodium salt (XTT; Sigma, Steinheim, Germany) plus coenzyme Qo (2,3-dimethoxy-5methyl-1,4-benzoquinone; Sigma), as described previously [31]. Although the XTT assay assesses the metabolic activity of cells, the results of the XTT assay are often used as a measure of cell damage [32,33]. In brief, immunosuppressive compounds were added in different concentrations either directly to 2 × 10^4^ dormand conidia (co-incubation for 17 hours) or to hyphae developed from 2 × 10^4^ conidia of the respective *Aspergillus* spp. (co-incubation for 6 or 17 hours), respectively. Fungi incubated alone served as control. Thereafter, hyphae were washed once with sterile aqua dest. and were incubated in XTT solution (0.25 mg/mL) supplemented with coenzyme Q_0_ (40 µg/mL) at 37 °C for another hour. The absorbance of the supernatant was assessed spectrophotometrically at 450 nm. Antifungal activity was calculated as follows: percentage of hyphal damage = (1−X/C) × 100, where X is the absorbance of experimental wells and C is the absorbance of control wells with hyphae only [32,33].

#### 4.3.3. Assessment of Fungal Cell Density

In order to evaluate the effect of the immunosuppressive compounds on the fungal growth of *Aspergillus*, fungal cell density was assessed spectrophotometrically [34,35,36,37]. Therefore, 2 × 10^4^ dormand conidia were incubated in the presence of immunosuppressive compounds in three different concentrations (CsA: 0.03, 0.15 and 0.75 µg/mL; MPA: 0.5, 5 and 50 µg/mL; mPRED: 0.025, 0.25 and 2.5 µg/mL) or alone for 17 hours in 200 µl YNB medium at 37 °C. Then, the optical density at 405 nm was assessed spectrophotometrically in a microplate photometer. 

### 4.4. Statistical Analyses

Data were analyzed using GraphPad Prism (version 5.04; GraphPad Software, La Jolla, CA, USA). Each concentration of the respective immunosuppressant was compared with the control only and therefore Student´s t-test was used. A two-sided P value of less than 0.05 was considered to be statistically significant.

## 5. Conclusions

The present data show that 1) MPA and CsA exhibit a species-specific inhibitory effect on the growth of *Aspergillus*, 2) higher fungal damage is observed when MPA and CsA are added to growing conidia than to mycelium and 3) mPRED increases the growth of *A. niger*, but has no major impact on the growth and viability of the other *Aspergillus* species tested. This study might increase the understanding of the complex interaction of immunosuppressants with various *Aspergillus* species and of the unique characteristics of each immunosuppressive agent (immunosuppression versus antifungal activity), which ultimately may have an impact to individualize immunosuppressive therapy.

## Supplementary Materials

The following are available online at https://www.mdpi.com/2076-0817/8/4/273/s1, Figure S1: Effect of immunosuppressive compounds on the viability of fully developed *Aspergillus* hyphae/mycelium after 17 hours of co-incubation.

## References

[1] Lehrnbecher T., Foster C., Vázquez N., Mackall C.L., Chanock S.J. (1997). Therapy-induced alterations in host defense in children receiving therapy for cancer. J. Pediatr. Hematol. Oncol..

[2] Vandevyver S., Dejager L., Tuckermann J., Libert C. (2013). New insights into the anti-inflammatory mechanisms of glucocorticoids: An emerging role for glucocorticoid-receptor-mediated transactivation. Endocrinology.

[3] Tramsen L., Schmidt S., Roeger F., Schubert R., Salzmann-Manrique E., Latge J.-P., Klingebiel T., Lehrnbecher T. (2014). Immunosuppressive Compounds Exhibit Particular Effects on Functional Properties of Human Anti-Aspergillus TH1 Cells. Infect. Immun..

[4] Allison A.C., Eugui E.M. (2000). Mycophenolate mofetil and its mechanisms of action. Immunopharmacology.

[5] Allison A.C. (2005). Mechanisms of action of mycophenolate mofetil. Lupus.

[6] Flores C., Fouquet G., Moura I.C., Maciel T.T., Hermine O. (2019). Lessons to Learn From Low-Dose Cyclosporin-A: A New Approach for Unexpected Clinical Applications. Front. Immunol..

[7] Miyakoshi S., Kusumi E., Matsumura T., Hori A., Murashige N., Hamaki T., Yuji K., Uchida N., Masuoka K., Wake A. (2007). Invasive Fungal Infection Following Reduced-Intensity Cord Blood Transplantation for Adult Patients with Hematologic Diseases. Biol. Blood Marrow Transplant..

[8] Fisher B.T., Robinson P.D., Lehrnbecher T., Steinbach W.J., Zaoutis T.E., Phillips B., Sung L. (2018). Risk Factors for Invasive Fungal Disease in Pediatric Cancer and Hematopoietic Stem Cell Transplantation: A Systematic Review. J. Pediatric Infect. Dis. Soc..

[9] Steinbach W.J., Schell W.A., Blankenship J.R., Onyewu C., Heitman J., Perfect J.R. (2004). In Vitro interactions between antifungals and immunosuppressants against Aspergillus fumigatus. Antimicrob. Agents Chemother..

[10] Li Y., Sun S., Guo Q., Ma L., Shi C., Su L., Li H. (2008). In Vitro interaction between azoles and cyclosporin A against clinical isolates of Candida albicans determined by the chequerboard method and time-kill curves. J. Antimicrob. Chemother..

[11] Kontoyiannis D.P., Marr K.A., Park B.J., Alexander B.D., Anaissie E.J., Walsh T.J., Ito J., Andes D.R., Baddley J.W., Brown J.M. (2010). Prospective Surveillance for Invasive Fungal Infections in Hematopoietic Stem Cell Transplant Recipients, 2001–2006: Overview of the Transplant-Associated Infection Surveillance Network (TRANSNET) Database. Clin. Infect. Dis..

[12] Baddley J.W., Marr K.A., Andes D.R., Walsh T.J., Kauffman C.A., Kontoyiannis D.P., Ito J.I., Balajee S.A., Pappas P.G., Moser S.A. (2009). Patterns of Susceptibility of Aspergillus Isolates Recovered from Patients Enrolled in the Transplant-Associated Infection Surveillance Network. J. Clin. Microbiol..

[13] Steinbach W.J., Singh N., Miller J.L., Benjamin D.K., Schell W.A., Heitman J., Perfect J.R. (2004). In Vitro interactions between antifungals and immunosuppressants against Aspergillus fumigatus isolates from transplant and nontransplant patients. Antimicrob. Agents Chemother..

[14] Cruz M.C., Fox D.S., Heitman J. (2001). Calcineurin is required for hyphal elongation during mating and haploid fruiting in Cryptococcus neoformans. EMBO J..

[15] Prokisch H., Yarden O., Dieminger M., Tropschug M., Barthelmess I.B. (1997). Impairment of calcineurin function in Neurospora crassa reveals its essential role in hyphal growth, morphology and maintenance of the apical Ca2+ gradient. Mol. Gen. Genet..

[16] Wong S.S.W., Rasid O., Laskaris P., Fekkar A., Cavaillon J.M., Steinbach W.J., Ibrahim-Granet O. (2017). Treatment of Cyclosporin A retains host defense against invasive pulmonary aspergillosis in a non-immunosuppressive murine model by preserving the myeloid cell population. Virulence.

[17] Punnett A., Sung L., Price V., Das P., Diezi M., Doyle J., Dupuis L.L. (2007). Achievement of target cyclosporine concentrations as a predictor of severe acute graft versus host disease in children undergoing hematopoietic stem cell transplantation and receiving cyclosporine and methotrexate prophylaxis. Ther. Drug Monit..

[18] Juvvadi P.R., Lee S.C., Heitman J., Steinbach W.J. (2017). Calcineurin in fungal virulence and drug resistance: Prospects for harnessing targeted inhibition of calcineurin for an antifungal therapeutic approach. Virulence.

[19] Oz H.S., Hughes W.T. (1997). Novel anti-Pneumocystis carinii effects of the immunosuppressant mycophenolate mofetil in contrast to provocative effects of tacrolimus, sirolimus, and dexamethasone. J. Infect. Dis..

[20] Husain S., Singh N. (2002). The impact of novel immunosuppressive agents on infections in organ transplant recipients and the interactions of these agents with antimicrobials. Clin. Infect. Dis..

[21] Ritter M.L., Pirofski L. (2009). Mycophenolate mofetil: Effects on cellular immune subsets, infectious complications, and antimicrobial activity. Transpl. Infect. Dis..

[22] Ng T.T.C., Robson G.D., Denning D.W. (1994). Hydrocortisone-enhanced growth of Aspergillus spp.: Implications for pathogenesis. Microbiology.

[23] Loose D.S., Stevens D.A., Schurman D.J., Feldman D. (1983). Distribution of a corticosteroid-binding protein in Candida and other fungal genera. J. Gen. Microbiol..

[24] Bellanger A., Minetos Y., Albert N., Shirazi F., Walsh T., Kontoyiannis D. (2015). Glucocorticosteroids do not impact directly growth rate and biomass of Rhizopus arrhizus (syn. R. oryzae) In Vitro. Virulence.

[25] Meier-Kriesche H.U., Friedman G., Jacobs M., Mulgaonkar S., Vaghela M., Kaplan B. (1999). Infectious complications in geriatric renal transplant patients: Comparison of two immunosuppressive protocols. Transplantation.

[26] Tharayil John G., Shankar V., Talaulikar G., Mathews M.S., Abraham Abraham M., Punnakuzhathil Thomas P., Korula Jacob C. (2003). Epidemiology of systemic mycoses among renal-transplant recipients in India. Transplantation.

[27] Narreddy S., Manavathu E., Chandrasekar P.H., Alangaden G.J., Revankar S.G. (2010). In Vitro interaction of posaconazole with calcineurin inhibitors and sirolimus against zygomycetes. J. Antimicrob. Chemother..

[28] Schwarz P., Schwarz P.V., Felske-Zech H., Dannaoui E. (2019). In vitro interactions between isavuconazole and tacrolimus, cyclosporin A or sirolimus against Mucorales. J. Antimicrob. Chemother..

[29] Rohatagi S., Barth J., Möllmann H., Hochhaus G., Soldner A., Möllmann C., Derendorf H. (1997). Pharmacokinetics of methylprednisolone and prednisolone after single and multiple oral administration. J. Clin. Pharmacol..

[30] Osunkwo I., Bessmertny O., Harrison L., Cheung Y.K., Van De Ven C., del Toro G., Garvin J., George D., Bradley M.B., Wolownik K. (2004). A pilot study of tacrolimus and mycophenolate mofetil graft-versus-host disease prophylaxis in childhood and adolescent allogeneic stem cell transplant recipients. Biol. Blood Marrow Transplant..

[31] Schmidt S., Tramsen L., Hanisch M., Latgé J.-P., Huenecke S., Koehl U., Lehrnbecher T. (2011). Human natural killer cells exhibit direct activity against Aspergillus fumigatus hyphae, but not against resting conidia. J. Infect. Dis..

[32] Moss B.J., Kim Y., Nandakumar M.P., Marten M.R. (2008). Quantifying metabolic activity of filamentous fungi using a colorimetric XTT assay. Biotechnol. Prog..

[33] Loures F., Levitz S. (2015). XTT Assay of Antifungal Activity. Bio-Protocol.

[34] Antachopoulos C., Demchok J.P., Roilides E., Walsh T.J. (2009). Fungal biomass is a key factor affecting polymorphonuclear leucocyte-induced hyphal damage of filamentous fungi. Mycoses.

[35] Antachopoulos C., Meletiadis J., Roilides E., Sein T., Sutton D.A., Wickes B.L., Rinaldi M.G., Merz W.G., Shea Y.R., Walsh T.J. (2006). Relationship between metabolism and biomass of medically important zygomycetes. Med. Mycol..

[36] Dannaoui E., Persat F., Monier M.-F., Borel E., Piens M.-A., Picot S. (1999). In-vitro susceptibility of Aspergillus spp. isolates to amphotericin B and itraconazole. J. Antimicrob. Chemother..

[37] Meletiadis J., Stergiopoulou T., O’Shaughnessy E.M., Peter J., Walsh T.J. (2007). Concentration-dependent synergy and antagonism within a triple antifungal drug combination against Aspergillus species: Analysis by a new response surface model. Antimicrob. Agents Chemother..

